# Managing Severe Proximal Femoral Fibrous Dysplasia in a Young Adult Using Hemiarthroplasty in a Resource-Constrained Healthcare System: A Case Report

**DOI:** 10.7759/cureus.117877

**Published:** 2026-10-13

**Authors:** Yustin Marinta, Fajar B Gardjito, Mujaddid Idulhaq

**Affiliations:** 1 Department of Orthopaedics and Traumatology, Prof. Dr. R. Soeharso Orthopaedic Hospital – Universitas Sebelas Maret, Solo, IDN

**Keywords:** bone diseases, developmental, femur, fibrous dysplasia of bone, hemiarthroplasty, resource-limited settings

## Abstract

Fibrous dysplasia of the proximal femur may lead to progressive deformity, pain, and pathological fractures, with shepherd's crook deformity representing one of its most severe manifestations. Although valgus osteotomy with intramedullary fixation is considered the preferred surgical treatment, extensive bone destruction and limited healthcare resources may preclude conventional reconstruction. We present a case of severe proximal femoral fibrous dysplasia successfully managed with bipolar cemented hemiarthroplasty in a resource-constrained healthcare setting. A 25-year-old man presented with progressive left hip pain, severe proximal femoral deformity, and impaired ambulation. Radiographic evaluation demonstrated lateral bowing of the proximal femur, while histopathological examination following core biopsy confirmed fibrous dysplasia. Internal fixation using an intramedullary nail was initially planned; however, intraoperative findings revealed extensive fibro-osseous replacement, severely compromised bone quality, and inadequate cortical support, making stable fixation technically impossible. Because more complex reconstructive procedures were not covered by the national health insurance system, a bipolar cemented hemiarthroplasty, augmented with plate fixation of the osteotomized fragments, was performed as a salvage procedure. The postoperative course was uneventful, with marked pain relief, improved gait, and satisfactory functional recovery at 10 months of follow-up (Oxford Hip Score 34/48). Conventional fixation for shepherd's crook deformity requires adequate residual cortical bone to achieve stable implant fixation. In cases with extensive cortical destruction and bone stock too thin to support reliable press-fit fixation, cemented hemiarthroplasty may represent a practical alternative. Preservation of the native acetabulum may also reduce postoperative instability while providing satisfactory functional outcomes. Bipolar cemented hemiarthroplasty may be an effective salvage option for selected patients with severe proximal femoral fibrous dysplasia when conventional fixation is technically unfeasible, particularly in resource-constrained healthcare systems.

## Introduction

Fibrous dysplasia (FD) is a benign tumor-like condition in which tissue of fibro-osseous origin replaces the normal bony structure. Similar to other benign bone lesions of the human skeleton, FD can be diagnosed incidentally or in association with certain symptoms [1]. Presenting symptoms might be pain, altered gait, fractures, and deformities. FD lesions can present in one bone (monostotic) or in multiple bones (polyostotic). The monostotic form is 1.5-2.5 times more common than the polyostotic variant and generally has a milder course, with less tendency to cause deformity [2].

The presence of the shepherd's crook deformity makes the diagnosis straightforward. This term is used to describe a pronounced coxa vara angulation and lateral bowing of the proximal femur. This deformity results in pain, restricted range of motion, limb shortening, leg length discrepancy, gait alteration, and a dramatically increased risk of pathologic proximal femur fractures. However, the treatment of shepherd's crook deformity is challenging because of the severely deformed and weakened bone [3,4].

The primary objectives of surgical treatment for shepherd's crook deformity are to restore adequate limb length and to re-establish sufficient abductor muscle tension, particularly of the gluteus medius, which is essential for a stable pelvis and an efficient, non-antalgic gait. Achieving both objectives simultaneously is technically demanding, since the same anatomical distortion responsible for the deformity - shortened femoral neck, altered femoral offset, and disruption of the abductor lever arm - is also the principal source of the operative difficulty encountered during reconstruction, including intraoperative judgement about implant sizing, soft-tissue balancing, and the risk of neurovascular stretch when correcting severe shortening.

Current surgical strategies include corrective valgus osteotomy, intramedullary fixation, biological reconstruction, total hip arthroplasty, curettage with strut graft, and proximal femoral replacement, depending on the extent of bone destruction and patient characteristics [2]. However, these procedures may not be feasible in resource-constrained healthcare systems because of implant availability and reimbursement limitations. In such settings, surgeons may need to adopt alternative reconstructive strategies that balance clinical effectiveness with healthcare accessibility.

## Case presentation

A 25-year-old man presented to the outpatient orthopaedic clinic with progressive left hip pain associated with severe deformity of the left proximal femur and difficulty walking. He had first noticed hip discomfort approximately two years earlier; over the preceding year, the pain and gait disturbance progressively worsened, resulting in an antalgic gait and significant functional limitation. Physical examination revealed a marked deformity of the left hip and proximal femur with restricted range of motion.

Plain radiographs (Figure 1) demonstrated lateral bowing of the proximal femur with an expansile lesion suggestive of fibrous dysplasia. To establish a definitive diagnosis, a core biopsy was performed, and histopathological examination demonstrated hard bone trabeculae with areas of cartilage and fibrous connective tissue stroma composed of monomorphic fibroblasts, with no evidence of malignancy. The findings were considered compatible with a benign mesenchymal lesion and were interpreted in conjunction with the clinical and radiological findings. Based on the clinical presentation, radiological characteristics, and histopathological findings, the lesion was diagnosed as fibrous dysplasia. Laboratory investigations, including serum alkaline phosphatase, calcium, and phosphate, were not available in our resource-limited setting; the diagnosis was therefore established on the basis of clinical, radiological, and histopathological findings alone, without clinical features suggestive of an associated endocrinopathy such as McCune-Albright syndrome.

**Figure 1:**
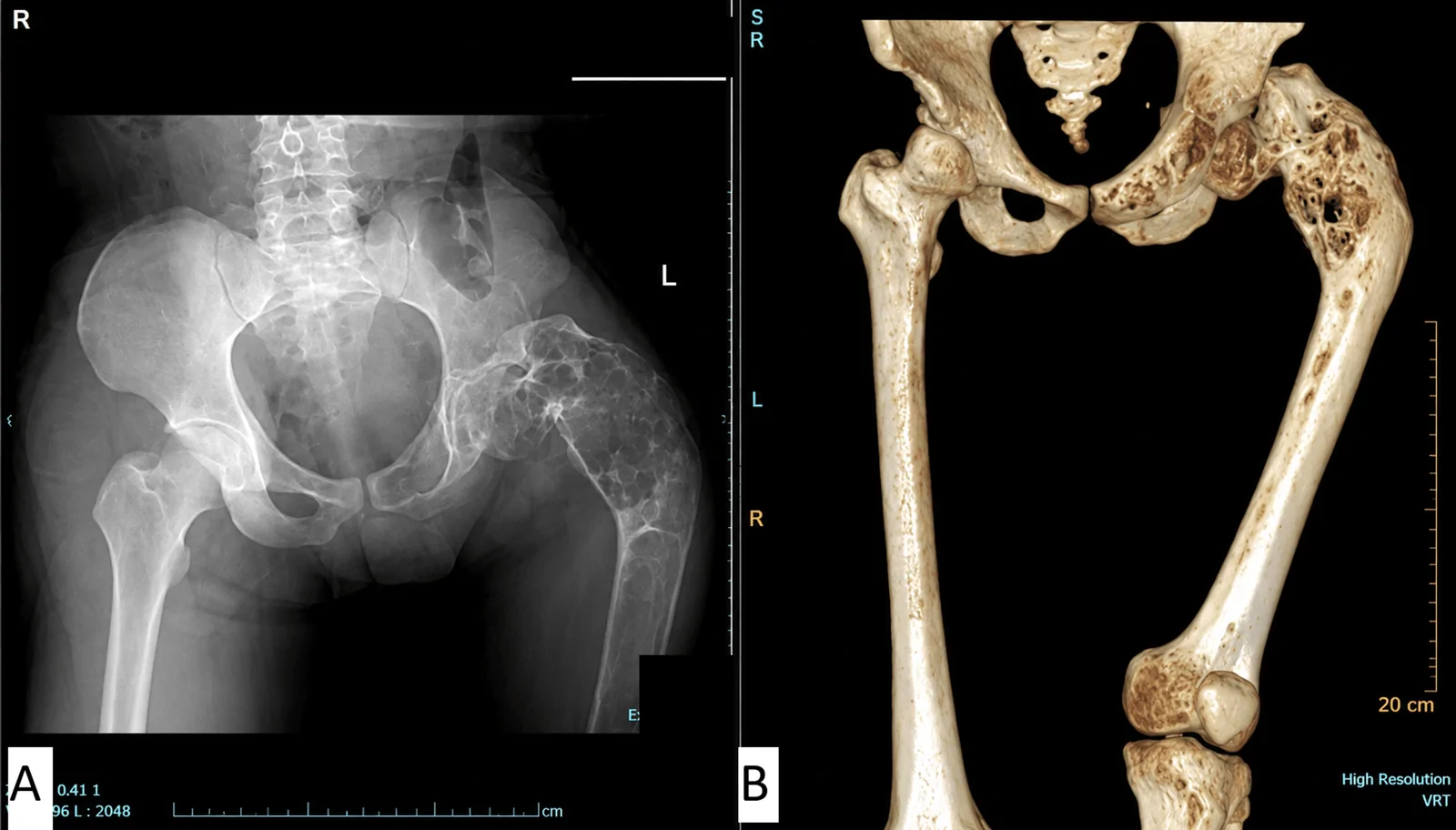
Preoperative imaging. (A) Preoperative plain radiograph demonstrating features consistent with fibrous dysplasia, including a ground-glass matrix, well-defined borders, and an intact but thinned cortex. (B) Three-dimensional computed tomography (CT) reconstruction demonstrating a well-defined expansile benign-appearing lesion with expansion and remodeling of the proximal femur from within the bone.

Considering the patient's persistent pain, progressive deformity, and impaired ambulation, surgical management was planned. The initial surgical strategy was internal fixation using an intramedullary nail. However, intraoperatively, the proximal femur was found to be extensively replaced by fibro-osseous tissue with severely compromised bone quality and inadequate cortical support, making stable fixation with an intramedullary implant technically unfeasible.

Under ideal circumstances, more complex reconstructive procedures or specialized implants might have been considered. Nevertheless, these options were not covered by the national health insurance system, creating significant financial limitations. Therefore, a cemented bipolar hemiarthroplasty (Figure 2) was performed as a salvage procedure to restore hip function while remaining within the available resources and insurance coverage.

**Figure 2:**
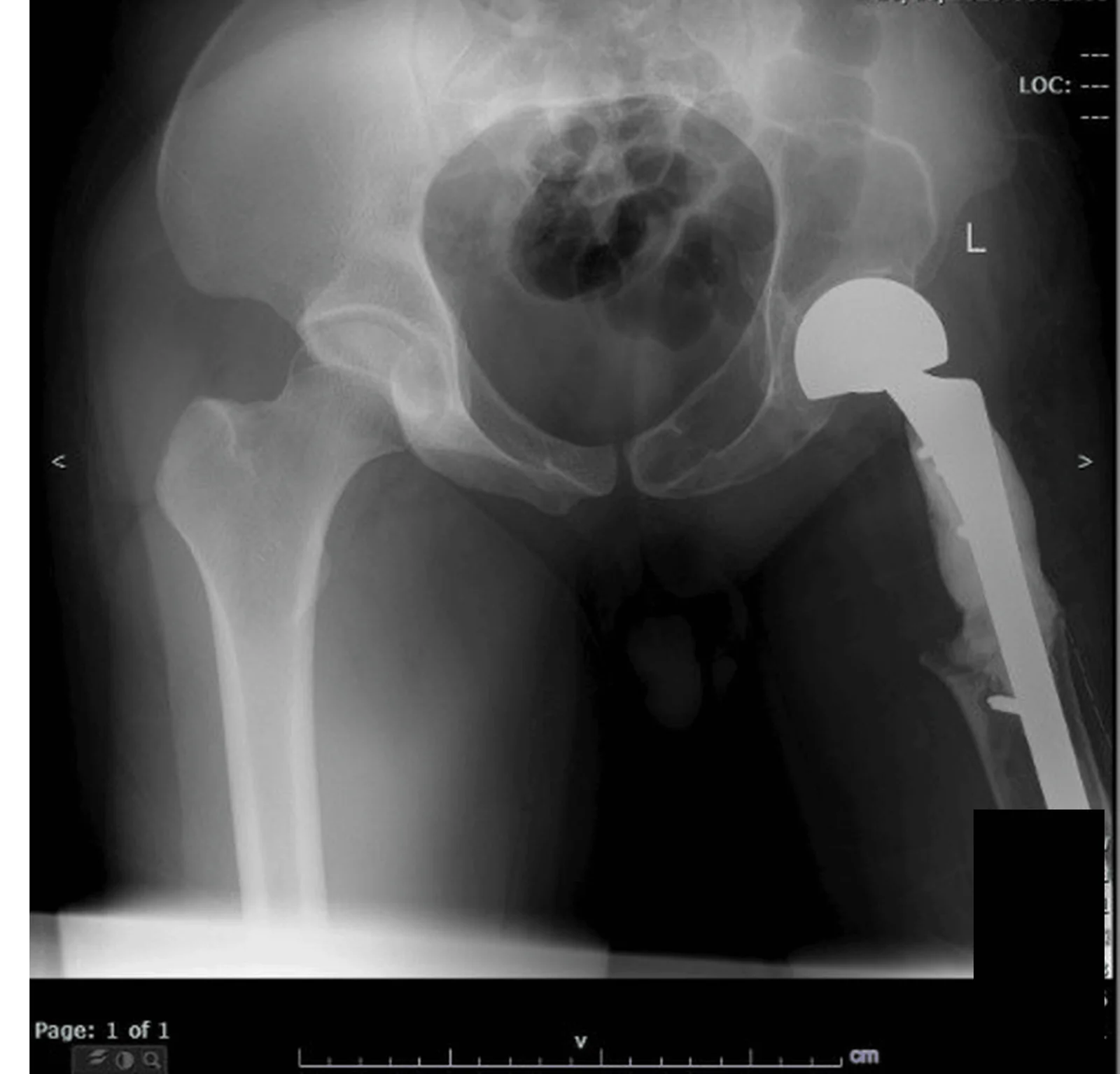
Immediate postoperative anteroposterior radiograph demonstrating bipolar cemented hemiarthroplasty.

Surgery was performed via a posterolateral approach to the proximal femur with the patient in the lateral decubitus position. The affected segment was confirmed fluoroscopically, following which multiple osteotomies of the proximal femur were performed; the resection level was reconfirmed before a wide excision of the diseased proximal femur was carried out. The residual femur was markedly thin and confirmed unsuitable for stable intramedullary nailing. The femoral canal was then medialized and reamed, and a trial femoral stem was inserted to assess fit and stability. A definitive cemented femoral stem (13 mm diameter, 220 mm length) was implanted with bone cement, and the osteotomized proximal femoral fragments were further stabilized around the stem with a locking plate and screws (open reduction and internal fixation). The hip was reduced using a 46 mm neutral bipolar head.

Limb length was assessed intraoperatively with reference to the contralateral limb. Postoperatively, the patient had a residual leg-length discrepancy of 3 cm, which was managed with observation and a shoe lift.

The postoperative course was uneventful. The patient experienced substantial pain relief and progressive improvement in gait. At approximately 10 months of follow-up (Figure 3), he reported a high level of satisfaction with the outcome, with improved mobility and the ability to perform daily activities independently (Figure 4). Functional outcome was assessed using the Oxford Hip Score, which was 34 out of 48 at final follow-up, indicating good functional recovery.

**Figure 3:**
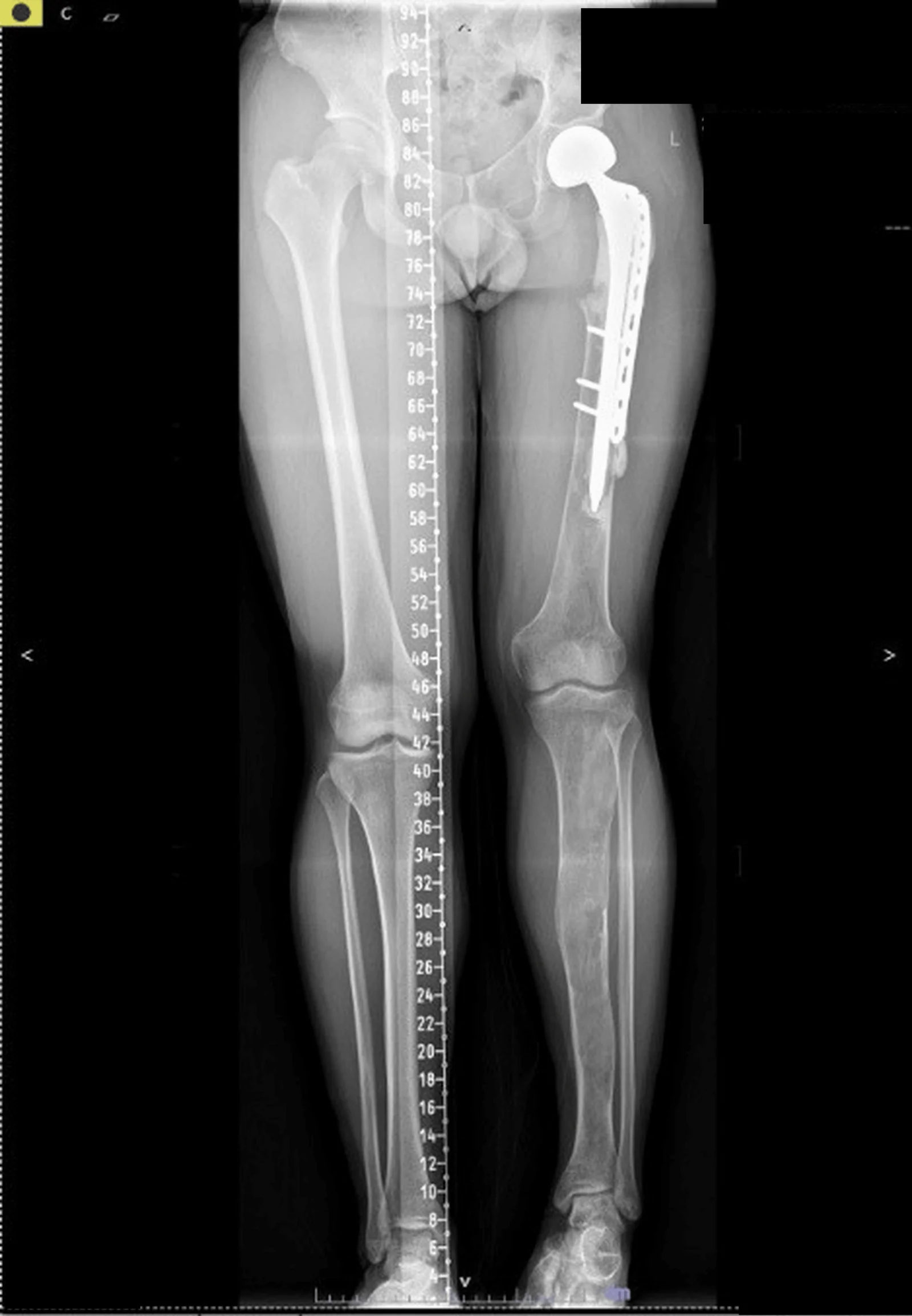
Ten months postoperative radiograph showing stable implant fixation without evidence of loosening or subsidence and residual leg-length discrepancy of 3 cm.

**Figure 4:**
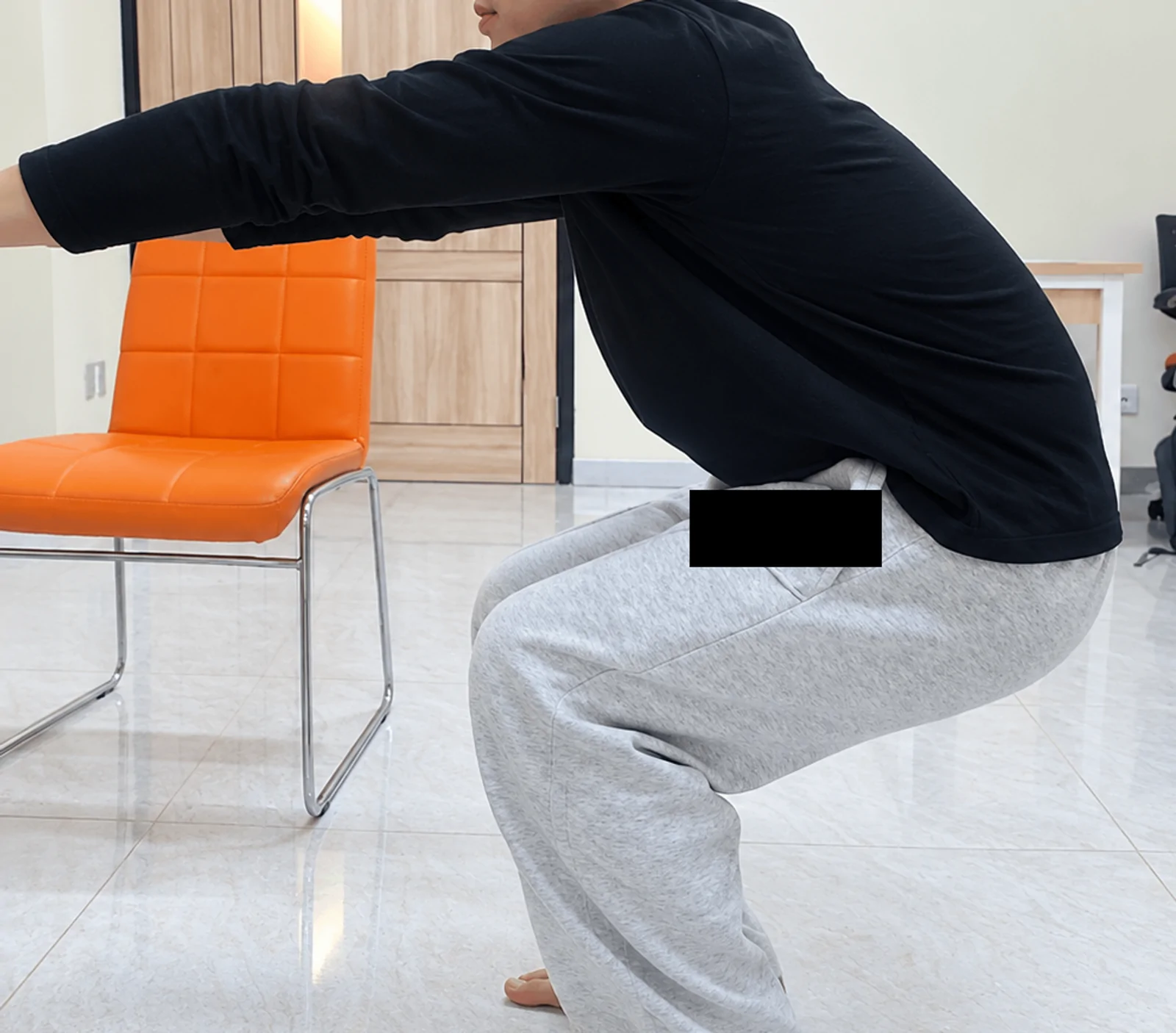
Clinical photograph at 10-months follow-up demonstrating painless squatting and satisfactory functional recovery. Written informed consent was obtained from the patient for the publication of this case report and the accompanying clinical photograph.

## Discussion

The management of fibrous dysplasia remains a challenge to the treating surgeon. The condition most commonly affects the long bones, which are weakened and are at risk of deformity. The proximal femur is especially at risk for deformation due to weight-bearing, resulting in progressive bowing and varus deformity, classically called the Shepherd's Crook deformity. The resulting deformity may manifest as limp in patients due to limb-length discrepancy. In progressive disease, it may cause painful hip and ultimately pathological fractures [5].

Fibrous dysplasia involving the proximal femur is frequently associated with progressive structural deformity due to abnormal bone remodeling, repetitive mechanical loading, and recurrent microfractures. These pathological processes may result in coxa vara, excessive femoral neck anteversion, anterior bowing, rotational deformity of the femoral shaft, and ultimately the characteristic shepherd's crook deformity [6]. Progressive deformity impairs hip biomechanics, leading to pain, gait disturbance, limb-length discrepancy, and an increased risk of pathological fracture. Early surgical intervention has been advocated, particularly in patients with polyostotic disease, to prevent further progression of deformity and preserve function [7].

Historically, curettage followed by cancellous bone grafting was commonly performed for fibrous dysplasia. However, long-term outcomes have demonstrated frequent graft resorption and persistence of the dysplastic lesion, limiting the effectiveness of this approach. Consequently, current surgical management focuses primarily on restoring mechanical alignment, correcting deformity, alleviating pain, and improving functional mobility rather than attempting complete eradication of the lesion [8]. Despite advances in surgical techniques, correction of shepherd's crook deformity remains technically demanding because of the complex three-dimensional deformity, compromised bone quality, and the difficulty of achieving stable fixation. Appropriate implant selection and meticulous surgical planning are therefore essential to obtain durable correction while minimizing operative complications [7].

Valgus osteotomy remains the preferred surgical procedure for correcting shepherd's crook deformity because it restores the femoral neck-shaft angle, re-establishes mechanical alignment, and reduces the risk of progressive deformity and pathological fracture. However, successful correction depends not only on the osteotomy itself but also on achieving stable internal fixation. Current evidence favors intramedullary fixation, particularly reconstruction nails with cephalocervical screws, because they provide longitudinal support along the femur while stabilizing the femoral neck. In contrast, plate-and-screw constructs are generally less suitable in fibrous dysplasia owing to poor bone quality, difficulty conforming to the deformed femoral anatomy, and an increased risk of stress concentration and recurrent deformity. Importantly, intramedullary fixation requires adequate residual cortical bone to provide sufficient implant purchase and mechanical stability. Extensive fibro-osseous replacement with cortical thinning may compromise fixation, resulting in inadequate support for the implant and increasing the risk of fixation failure. Consequently, when sufficient cortical bone cannot be preserved, conventional valgus osteotomy with internal fixation may no longer be feasible, necessitating consideration of alternative reconstructive procedures [9].

Proximal femoral replacement is broadly classified into two reconstructive modalities: hemiarthroplasty (HA), which replaces the proximal femur while preserving the native acetabulum, and total hip arthroplasty (THA), which includes replacement of both the femoral and acetabular components [10].

Beyond implant selection, the present case illustrates several practical difficulties encountered when performing hemiarthroplasty in severely dysplastic bone. Intraoperative assessment confirmed that the proximal femoral cortex was too thin to support stable intramedullary nailing, which led to wide excision of the diseased segment through multiple osteotomies rather than an attempt at corrective valgus osteotomy alone. Because the residual bone stock was judged insufficient to achieve reliable primary fixation with a press-fit stem, a cemented femoral component was used instead to provide immediate mechanical stability independent of bone ingrowth; the osteotomized proximal fragments were further secured around the stem with a locking plate and screws to protect the reconstruction during early healing. Intraoperative limb length restoration was guided by comparison with the contralateral limb; a residual leg-length discrepancy of 3 cm remained postoperatively, which was managed nonoperatively with observation and a shoe lift, as it was considered within a functionally tolerable range. Preservation of the native acetabulum, rather than proceeding to total hip arthroplasty, simplified the reconstruction by removing the need for acetabular component fixation in already compromised bone stock, consistent with reports that hemiarthroplasty is associated with lower dislocation and infection rates than total hip arthroplasty in similar reconstructive settings [11,12].

Although THA may be indicated when the acetabulum is involved or demonstrates advanced degenerative changes, HA is generally preferred when the acetabulum is preserved. In orthopedic oncology, Salvini et al. reported that HA has been associated with lower rates of infection and dislocation while achieving functional outcomes comparable to THA. Preservation of the native acetabulum may enhance joint stability and avoid complications related to acetabular component implantation [11]. Banskota et al. also reported that HA demonstrated a lower dislocation rate than THA following proximal femoral reconstruction, likely due to preservation of the native acetabulum and greater joint stability [12]. Therefore, in our patient, who had an intact acetabulum and severe proximal femoral destruction secondary to fibrous dysplasia, bipolar cemented hemiarthroplasty represented a reasonable reconstructive option. Furthermore, in our resource-limited setting, this approach was more feasible because a proximal femoral replacement with hemiarthroplasty was covered by the national health insurance system, whereas total hip arthroplasty was limitedly covered.

This case highlights that surgical decision-making should be individualized according to both anatomical feasibility and healthcare resource availability. Although valgus osteotomy with intramedullary fixation remains the preferred treatment for shepherd's crook deformity, surgeons may need to adopt alternative reconstructive strategies when adequate fixation cannot be achieved, or ideal implants are inaccessible. In conclusion, bipolar cemented hemiarthroplasty, augmented with plate fixation of the osteotomized fragments, may represent a feasible salvage option for selected patients with severe proximal femoral fibrous dysplasia when conventional fixation is not technically achievable. This case underscores the importance of individualized surgical planning that considers both anatomical feasibility and healthcare resource availability.

Limitations

This report has several limitations. Laboratory and endocrine work-up (e.g., serum alkaline phosphatase, calcium, and phosphate) were not available in our resource-limited setting, and the diagnosis relied on clinical, radiological, and histopathological findings alone. The follow-up period of approximately 10-12 months is relatively short to establish the long-term durability of hemiarthroplasty in a young patient, and does not allow assessment of outcomes such as acetabular erosion, implant loosening or subsidence, periprosthetic fracture, or the eventual need for revision surgery, all of which are of particular concern given the patient's young age and expected long-term implant demand. Although functional outcome was assessed using a validated hip-specific instrument (Oxford Hip Score, 34/48 at final follow-up), longer-term follow-up with serial radiographic surveillance and repeated validated scoring is recommended to better characterize the durability of this salvage procedure.

## Conclusions

Severe proximal femoral fibrous dysplasia with shepherd's crook deformity presents a significant reconstructive challenge because compromised bone quality may preclude stable internal fixation despite valgus osteotomy being the preferred treatment. In our patient, extensive fibro-osseous replacement and inadequate cortical support made conventional fixation technically impossible. Bipolar cemented hemiarthroplasty, combined with plate fixation of the osteotomized proximal femoral fragments, provided a successful salvage procedure, resulting in substantial pain relief, restoration of ambulation, and satisfactory functional recovery (Oxford Hip Score 34/48) at 10 months of follow-up. This case highlights the importance of individualized surgical decision-making based not only on anatomical feasibility but also on healthcare resource availability, suggesting that hemiarthroplasty may be a practical alternative when standard reconstructive options are technically unfeasible or inaccessible in resource-constrained settings.
